# Proteomics coupled with *in vitro* model to study the early crosstalk occurring between newly excysted juveniles of *Fasciola hepatica* and host intestinal cells

**DOI:** 10.1371/journal.pntd.0010811

**Published:** 2022-10-12

**Authors:** David Becerro-Recio, Judit Serrat, Marta López-García, Javier Sotillo, Fernando Simón, Javier González-Miguel, Mar Siles-Lucas

**Affiliations:** 1 Parasitology Unit, Institute of Natural Resources and Agrobiology of Salamanca (IRNASA-CSIC), Salamanca, Spain; 2 Parasitology Reference and Research Laboratory, Centro Nacional de Microbiología, Instituto de Salud Carlos III, Majadahonda, Madrid, Spain; 3 Laboratory of Parasitology, Faculty of Pharmacy, University of Salamanca, Salamanca, Spain; 4 Molecular Parasitology Laboratory, Centre of One Health (COH), Ryan Institute, National University of Ireland, Galway, Ireland; Universidad de la Republica, Uruguay, URUGUAY

## Abstract

Fasciolosis caused by the trematode *Fasciola hepatica* is a zoonotic neglected disease affecting animals and humans worldwide. Infection occurs upon ingestion of aquatic plants or water contaminated with metacercariae. These release the newly excysted juveniles (FhNEJ) in the host duodenum, where they establish contact with the epithelium and cross the intestinal barrier to reach the peritoneum within 2–3 h after infection. Juveniles crawl up the peritoneum towards the liver, and migrate through the hepatic tissue before reaching their definitive location inside the major biliary ducts, where they mature into adult worms. Fasciolosis is treated with triclabendazole, although resistant isolates of the parasite are increasingly being reported. This, together with the limited efficacy of the assayed vaccines against this infection, poses fasciolosis as a veterinary and human health problem of growing concern. In this context, the study of early host-parasite interactions is of paramount importance for the definition of new targets for the treatment and prevention of fasciolosis. Here, we develop a new *in vitro* model that replicates the first interaction between FhNEJ and mouse primary small intestinal epithelial cells (MPSIEC). FhNEJ and MPSIEC were co-incubated for 3 h and protein extracts (tegument and soma of FhNEJ and membrane and cytosol of MPSIEC) were subjected to quantitative SWATH-MS proteomics and compared to respective controls (MPSIEC and FhNEJ left alone for 3h in culture medium) to evaluate protein expression changes in both the parasite and the host. Results show that the interaction between FhNEJ and MPSIEC triggers a rapid protein expression change of FhNEJ in response to the host epithelial barrier, including cathepsins L3 and L4 and several immunoregulatory proteins. Regarding MPSIEC, stimulation with FhNEJ results in alterations in the protein profile related to immunomodulation and cell-cell interactions, together with a drastic reduction in the expression of proteins linked with ribosome function. The molecules identified in this model of early host-parasite interactions could help define new tools against fasciolosis.

## Author summary

Fasciolosis caused by *Fasciola hepatica* poses a serious concern for animal and human health worldwide. Treatment and prevention of this disease is challenging due to the appearance of parasites that are resistant to the current treatment and the suboptimal efficacy of assayed vaccines. In this context, the importance of early interactions with the host for parasite migration and infection progression prompted us to develop and study new models of interaction between *F*. *hepatica* juveniles and their host. Here, an *in vitro* model coupled with a quantitative proteomics approach was developed to evaluate the changes in *F*. *hepatica* newly excysted juveniles right after their contact with intestinal epithelial cells, which represent the first line of host cells that the parasite encounters upon host infection. Additionally, this model has been used to decipher potential changes elicited by the parasite in the host intestine. A proteomic study of parasites and host cells upon co-incubation revealed specific changes in the repertoire of proteins from both host and parasite following their interaction. These proteins could be of importance for parasite migration and survival inside the host, and give new important insights to understand the host-parasite molecular crosstalk in early fasciolosis.

## Introduction

Early host-parasite interactions are of paramount importance in the context of helminth infections as these mechanisms occur during the first contact between both organisms and the resulting molecular crosstalk determines the overall success of the parasite invasion process [1]. From a clinical point of view, knowledge of this initial infection stage is essential for diagnosis and treatment, as an early detection of infection would allow for the disease to be managed more effectively and before the parasite is able to trigger immune evasion mechanisms that favour its establishment in a niche that is poorly accessible to the host immune response. *In vitro* models to dissect the early infection stages of parasitic infections constitute a valuable tool that has been successfully implemented for the study of different endoparasites of medical and veterinary interest [2,3]. Moreover, the usefulness of these models has been substantially enhanced by the introduction of proteomic and transcriptomic tools [4], which facilitate the analysis of the host-parasite molecular repertoire and offer a powerful resource for the identification of therapeutic targets.

*Fasciola hepatica* is the causative agent of fasciolosis, a foodborne parasitic disease responsible for enormous economic losses in the livestock industry (mainly affecting large-size herbivorous species) of around $3 billion per year [5], as well as a major human health problem in several endemic regions around the planet [6,7]. The first contact with the definitive host takes place in the small intestine after ingestion of food or water contaminated with metacercariae, which excyst in the duodenum and release the newly excysted juveniles (FhNEJ). These are able to cross the intestinal wall and reach the peritoneum in approximately 3 h, and from this point continue their migratory cycle towards the liver [8,9]. Although the migratory phases that appear after crossing of the intestinal wall and which are responsible for the acute phase of the disease have attracted significant research interest over the past decades, parasite interaction with the intestinal epithelium itself has received little attention and, in fact, the body of knowledge about the molecular responses triggered by the interaction between FhNEJ and the intestinal epithelium is scarce [10]. In this context, molecular characterisation of host-parasite interactions at the intestinal level could provide new biomarkers for early diagnosis and treatment of fasciolosis as well as potential vaccine candidates against this disease [11].

Several experimental models have been developed over the past decades to study the passage through the intestinal wall of FhNEJ based on reproducing this migration step by using sections of the rat jejunum under *ex vivo* [12] and *in vitro* conditions [13]. Although these studies provided valuable information on the path followed by *F*. *hepatica* juveniles during migration, they did not emphasise the molecular events that occur in both the parasite and the host in response to their first interaction. Previously, our group developed an *in vitro* host-parasite interaction model aimed at reproducing the contact of FhNEJ with the host’s intestinal epithelium by co-culturing these parasites with mouse primary small intestinal epithelial cells (MPSIEC). In this model, proteomic changes induced by host-parasite interactions within the first 24 h of contact were assessed using Isobaric Tag for Relative and Absolute Quantitation (iTRAQ) [14].

In the present study, we employed this *in vitro* system to address protein expression changes in both FhNEJ and MPSIEC after a short and biologically-relevant interaction time of 3 h, followed by proteomic analyses of all samples using a new era quantitative proteomic approach known as Sequential Window Acquisition of All Theoretical Mass Spectra (SWATH-MS). This approach comprises a label-free identification and quantification methodology, which allows for improved accuracy and reproducibility [15] by computing mass spectra in multiple windows of a few *m/z* units. The ultimate goal is to be able to identify the proteins playing a key role in the first host-parasite contact in fasciolosis, as well as exploring the potential molecular interactions and relevant biological processes involved in the early phase of the infection.

## Materials and methods

### Primary intestinal epithelial cell culture

Mouse small intestinal epithelial cells (C57BL/6 MPSIEC) from Cell Biologics (ref. C57-6051) were grown and expanded as previously described [14]. Briefly, cells were plated in petri dishes precoated with 0.2% porcine gelatine, and cultured in epithelial cell growth medium (Innoprot) at 37°C in a humidified atmosphere in the presence of 5% CO_2_. Medium was replaced every 24–48 h and when confluence was reached (approximately every 48 h), cells were split in a 1:3 ratio.

### *F*. *hepatica* metacercariae *in vitro* excystment

*F*. *hepatica* metacercariae (Italian strain) were purchased from Ridgeway Research LTD (UK) and *in vitro* excystment was performed as previously described [16]. In brief, CO_2_ was bubbled for 30 sec in a tube containing 10 ml of distilled water, and sodium hydrosulphite (Sigma) was added to a final concentration of 0.02 M. This solution was added to metacercariae and incubated for 1 h at 37°C. Then, the metacercariae were washed twice with warm distilled water, resuspended in 5 ml of Hank’s balanced salt solution (Sigma) supplemented with 0.03 M HEPES (Sigma) and 10% lamb bile (from a local abattoir) and incubated at 37°C for 4–5 h. FhNEJ were manually collected with a micropipette, washed twice with sterile PBS and immediately incubated with host cells.

### *In vitro* interaction model

The *in vitro* interaction model used in this study was previously described by González-Miguel *et al*. [14], with some modifications. Briefly, MPSIEC on passage 5 were cultured in 60 mm gelatin-coated plates (n = 3) until cell confluence was reached, and they were stimulated with 400 FhNEJ per plate. The co-culture was incubated for 3 h at 37°C in a 5% CO_2_ atmosphere, after which MPSIEC and FhNEJ were separated by successive washes with sterile PBS. Additionally, 3 batches of 400 FhNEJ and 3 confluent plates of passage 5 MPSIEC were incubated under the same conditions without stimulation, consisting of MPSIEC and FhNEJ left alone for 3 h in the same experimental conditions that co-cultured cells and FhNEJ, and were used as negative controls. Number of cells (3.2 x 10^6^ intestinal epithelial cells per plate) and number of FhNEJ for *in vitro* stimulation experiments was settled to maximize the chances to have the majority of cells in contact with the FhNEJ and thus properly stimulated and to obtain enough stimulated host and parasite material for downstream analyses, although this specific host cells/parasite ratio could not reflect the *in vivo* situation.

### Protein extraction

All FhNEJ samples were treated as described by García-Campos *et al*. [17] for isolation of the tegument and somatic fractions. This protocol is based on the procedure described earlier in [18], with minor modifications, in which the tegumental nature of the antigens extracted from *F*. *hepatica* intact worms was tested by the peroxidase-antiperoxidase immunocytochemical technique at the light microscope level [18]. Thus, and although a complete separation of tegument and somatic FhNEJ extracts is expected to be difficult to achieve due to technical constraints, enriched protein fractions of the tegument can be obtained as described by other authors [17, 18]. FhNEJ were washed twice with sterile PBS, resuspended in an appropriate volume of PBS + 1% Nonidet P-40 (Sigma) and incubated at room temperature with gentle stirring for 30 min in order to extract the tegument fraction. After incubation, the samples were centrifuged (300 *g*, 5 min) and the supernatant of the detergent-soluble extract containing the enriched protein fraction of the FhNEJ tegument was transferred to clean tubes. The pellet containing naked FhNEJ was resuspended in RIPA buffer (Sigma) and disrupted by ultrasound (5 cycles of 30 s). Finally, samples were centrifuged (1,000 *g*, 5 min) and the clarified supernatant containing somatic proteins was transferred to clean tubes. All *F*. *hepatica* samples were treated with protease inhibitors and stored at -80°C until use.

Cell culture plates containing MPSIEC were washed twice with sterile PBS and incubated on ice for 10 min to weaken cell adhesion. Cells were carefully lifted from the plate using a cell scraper, collected with a pipette tip and deposited into 1.5 ml tubes. Cytosolic and membrane proteins were separately isolated using the Mem-PER Plus Membrane Protein Extraction kit (Thermo Fisher) following the manufacturer’s instructions. Protease inhibitors were added at the appropriate ratio and the samples were stored at -80°C until use.

### Proteomic analysis

Quantitation of all protein samples was performed using a detergent-compatible kit (Protein Quantification Assay; Machery-Nagel) following the manufacturer’s instructions. In-gel digestion of proteins was carried out in accordance to the described protocols [19] with the following modifications: 20 μg of each sample were dissolved in 20 μl of Laemmli Sample Buffer (Bio-Rad) supplemented with β-mercaptoethanol and heated for 5 min at 95°C, after which they were loaded onto an Any kD precast 1D PAGE gel (Bio-Rad) and run at 200 V for 5 min. After separation, samples were fixed with 40% ethanol/10% acetic acid and stained with colloidal Coomasie.

Gel bands were cut into pieces and reduction of peptides was performed using 10 mM dithiothreitol (DTT) in 50 mM of ammonium bicarbonate (20 min at 60°C, 850 rpm), and for alkylation 55 mM iodoacetamide in 50 mM ammonium bicarbonate (30 min at room temperature in the dark, 850 rpm) were used. Trypsin digestion was performed using a 1:25 ratio (ng enzyme/μg protein) in 50 mM ammonium bicarbonate (overnight at 37°C with no stirring), and subsequent extraction of peptides was carried out using pure acetonitrile, which was dried in a rotary evaporator at 37°C. Finally, peptides were resuspended in 2% ACN, 0.1% TFA (5 min at room temperature, 850 rpm). For library construction, all *F*. *hepatica* samples were pooled together and loaded onto an analytical column (LC Column, 3 μ C18-CL, Nikkyo) equilibrated in 5% ACN/0.1% formic acid. Elution was performed over 120 min in a linear gradient of 5–35% solvent B (A: 0.1% FA; B: ACN, 0.1% FA) at a flow rate of 300 nl/min and analysed in a mass spectrometer nanoESI qQTOF (5600 TripleTOF, ABSCIEX).

Analysis was carried out in a data-dependent mode (DDA). Survey MS1 scans were acquired from 350–1250 *m/z* for 250 ms, whereas the quadrupole resolution was set to ‘UNIT’ for MS2 experiments, which were acquired 100–1500 *m/z* for 150 ms in high sensitivity mode. *Mus musculus* samples were treated using the same workflow in order to generate a mouse-specific peptide library. Individual samples were acquired with the tripleTOF operating in SWATH mode (DIA), in which a 0.050-s TOF MS scan from 350–1250 *m/z* was performed, followed by 0.080-s product ion scans from 350–1250 *m/z* on the 32 defined windows (3.05 sec/cycle).

Top 100 were used in DDA mode. In DIA mode, the quadrupole was operated using ‘Unit’ resolution, using 15 Da windows with 1 Da overlap between windows. Collision energy was determined using the following equation: CE = (Slope) * x * (*m/z*) + intercept. Slope = 0.625 and intercept = -3 coefficients were used, and all peptides were fragmented as z = 2. MS/MS scans were performed using a resolution of 15000, and no precursors were selected, as they all entered the collision chamber and were fragmented as z = 2 charge. No technical replicates were used. Instead of that, reproducibility was evaluated with a quality control of 3 injections of 500 ng K562.

The .*wiff* files corresponding to the peptide libraries were processed via Protein Pilot v5.0 (SCIEX) to generate a peak list used as reference for individual sample quantitation. The Paragon algorithm [20] was applied to reference databases with the following parameters: trypsin specificity, iodoacetamide cys-alkylation and taxonomy not restricted. Only proteins with at least 2 identified peptides and < 1% FDR were considered for subsequent analysis. The databases used in this study (downloaded the 25/02/2020) contained the predicted proteome of *F*. *hepatica* (PRJEB25283, https://parasite.wormbase.org/Fasciola_hepatica_prjeb25283/Info/Index) or the proteome of *Mus musculus* (https://www.uniprot.org/proteomes/UP000000589), both appended to the cRAP contaminant database (https://www.thegpm.org/crap/).

The .*wiff* files obtained from SWATH individual acquisitions were analysed using PeakView 2.1 (SCIEX) and MarkerView 3.0 (SCIEX). Protein areas were calculated and normalized to the total sum of the areas of all the quantified proteins, and proteins identified within the contaminant database were removed from the dataset prior to the differential expression analyses.

The mass spectrometry proteomics data have been deposited to the ProteomeXchange Consortium via the PRIDE [21] partner repository with the dataset identifier PXD033952.

The proportion of proteins showing transmembrane domains in the proteins identified in both the membrane and the cytosol fractions of MPSIEC was evaluated with the DeepTMHMM tool (https://dtu.biolib.com/DeepTMHMM). Additionally, the presence/absence of the membrane marker COX-IV protein was assessed in both extracts.

### Statistical and computational analysis

Log2 of each detected protein was determined prior to quantitative analysis. Statistical treatment was performed using GraphPad Prism 9.0.1, and evaluation of differences between stimulated and control samples was performed using *Student’s t-test* coupled with Benjamini, Krieger and Yekutieli *post-hoc* corrections. Only proteins with a q-value <0.05 were considered as differentially expressed. Principal Component Analysis (PCA) of all samples was performed with the online tool ClustVis [22], and the Volcano plots showing the distribution of up- and down-regulated proteins were obtained using the *ggplot2* package of R software.

Functional annotation of the differentially expressed proteins was performed with Blast2GO 5.2 using the blastp and InterProScan algorithms to obtain protein descriptions against the NCBI and EBI databases, respectively. Gene Ontology (GO) terms in the Biological Process (BP), Molecular Function (MF) and Cellular Component (CC) categories were computed, and GOs obtained by both algorithms were merged.

GO terms within the BP and MF categories were parent removed and represented using the ReViGO tool (http://revigo.irb.hr/), showing the size of each term in proportion to its Nodescore. Differentially expressed proteins from MPSIEC samples were represented in interaction networks using STRING (https://string-db.org/).

## Results and discussion

Prior to invading host tissues, FhNEJ must interact with cells of the intestinal epithelium, which comprise the first physical barrier and the first line of innate immune defence encountered by the parasite [23]. Consequently, in this study we aimed to reproduce the physical and biological interactions between the juvenile worms of *F*. *hepatica* and the cells of the intestinal epithelium in an *in vitro* model, and study the proteomic changes induced during the first 3 h of interaction to shed light on host-parasite relationships occurring before the ’point of no return’ in fasciolosis is reached.

**Fig 1:**
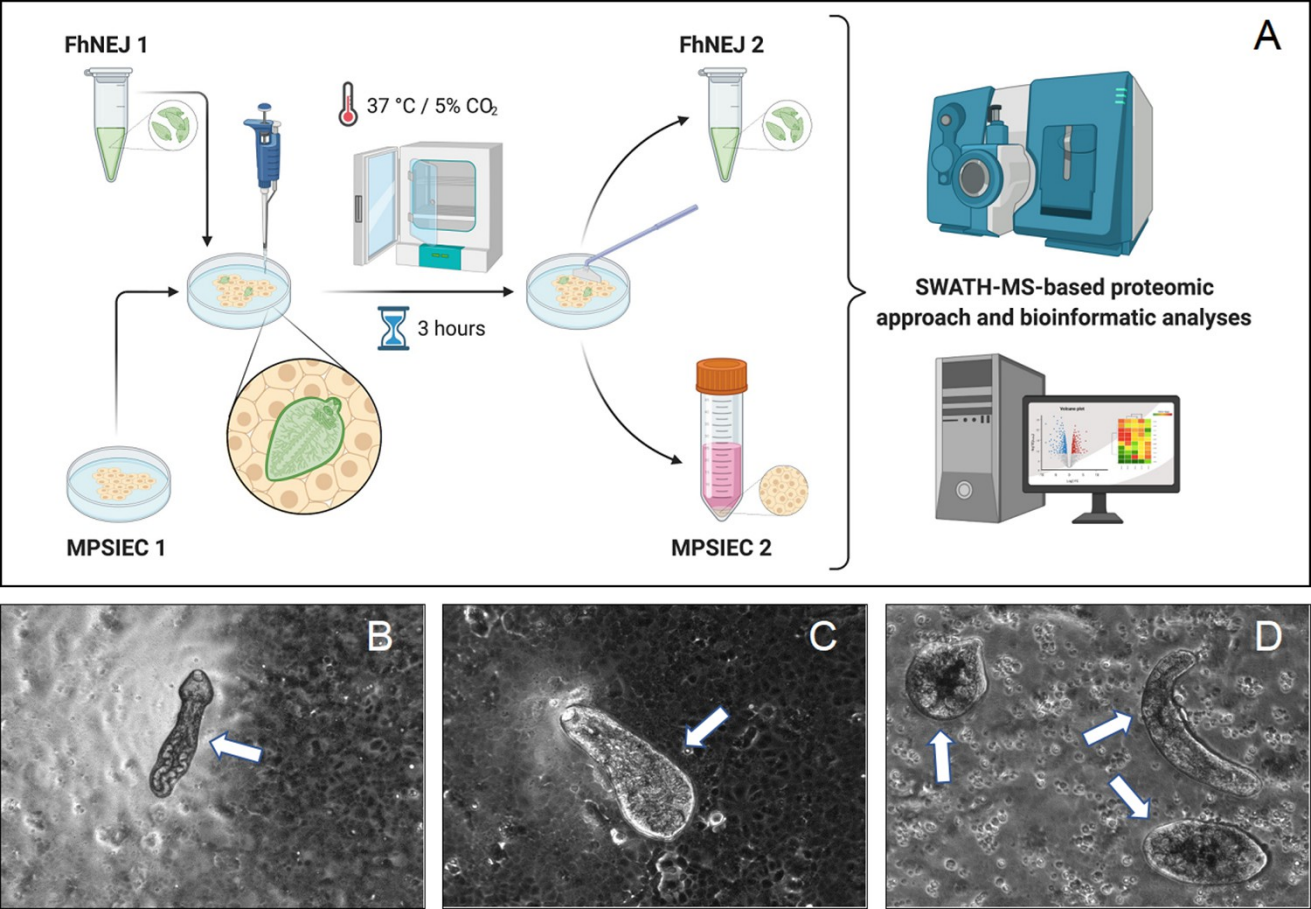
Experimental design of the *in vitro* interaction model between *F*. *hepatica* juveniles (FhNEJ) and mouse intestinal epithelial cells (MPSIEC). (A) Parasitic extracts and host cell lysates were obtained before (FhNEJ 1, MPSIEC 1) and after co-culturing for 3 h (FhNEJ 2, MPSIEC 2) and their proteins analyzed by SWATH-MS. (B, C, D) Representative images of MPSIEC (at the background of each image) stimulated with the FhNEJ (arrows) are shown (optical microscope, 10x). Panel A created with BioRender.com.

The experimental design that we proposed is based on co-culturing FhNEJ with MPSIEC for 3 h at 37°C, a time frame that is similar to that required by FhNEJ to migrate through the host intestinal wall in a physiological setting (Fig 1). After establishing contact with the mouse intestinal cell monolayer, FhNEJ actively displayed their typical locomotion process, which is based on a smooth sequence of whole-body waves coordinated with alternate attachment and release of the oral and ventral suckers and a vermiform peristalsis [24,25] (Fig 1, S1 Video). After 3 h of co-culture, both FhNEJ and epithelial cells were separated and subjected to protein extraction from two compartments: detergent-soluble extract enriched with tegument and soma in the case of FhNEJ, and cytosol and membrane for mouse cells. Thereby, we aimed to isolate the proteins comprising the host-parasite interface. It is worth mentioning that the tegument of *F*. *hepatica* includes a biologically active and metabolically complex matrix that is continuously exposed to host tissues, whose dynamic composition is directly related to the changing environment that the juvenile parasite encounters during its migration process [26–28]. For that reason, the FhNEJ tegument has been proposed as a potential source of therapeutic targets against fasciolosis [10]. Nevertheless, it should be mentioned that our detergent-soluble extract could contain components of the FhNEJ tegument, together with components of other areas of FhNEJ that could be exposed to the extraction protocol.

### Protein identification and quantification

After establishing the *in vitro* interaction model, we applied a SWATH-MS-based proteomic approach [29] in order to identify protein expression changes in both the parasite and host cells upon their interaction. Despite its robustness in comparing protein abundance of different samples, this technique has been infrequently used in parasitology research. In our study, the application of SWATH-MS analysis resulted in a total of 6,169 and 24,965 spectra identified in FhNEJ and MPSIEC samples, respectively, corresponding to 541 and 2,012 proteins detected with at least 2 peptides using an FDR below 1%. From the identified proteins, 505 and 427 were present in all replicates from the detergent-soluble extract enriched with tegument and soma extract of FhNEJ, respectively, and 1,497 and 1,508 proteins were found in all replicates from the cytosol and membrane extracts of MPSIEC, and could therefore be quantified. Common contaminant proteins represented 31 and 33 of the proteins identified in the tegument and soma of FhNEJ, respectively, and 134 and 177 contaminant proteins, respectively, were detected in the cytosol and membrane of MPSIEC and were excluded from further statistical analysis. After contaminant removal, a total of 474 and 394 common proteins were identified in all FhNEJ tegument and soma samples, respectively. This represents a 4.87% and 4.05% of the predicted *F*. *hepatica* proteome (9732 proteins). As for mouse, 1363 were identified in all cytosol samples whereas 1331 proteins were listed in the membrane fraction, which represents a 2.46% and a 2.41% of the *Mus musculus* theoretical proteome (55342 proteins).

**Fig 2:**
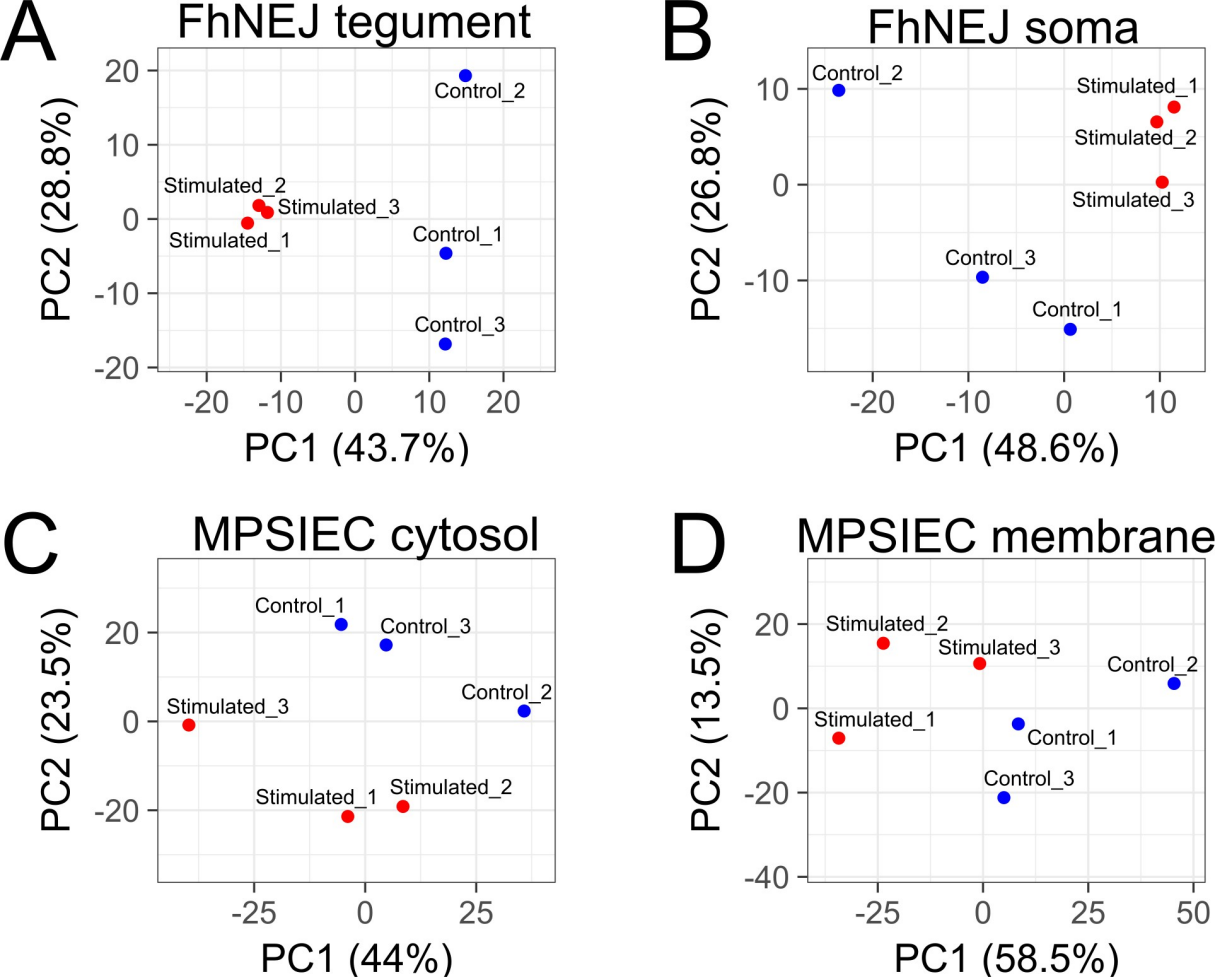
Principal Component Analysis (PCA) of each group of samples analysed in the study. PCA analyses of FhNEJ detergent-soluble extract enriched with tegument (FhNEJ tegument; A), FhNEJ somatic (B), MPSIEC cytosol (C) and MPSIEC membrane (D) protein extracts. Blue dots represent the three control replicates (incubated alone for 3h; control) whereas red dots represent the three replicates co-incubated for 3h (stimulated). The percentage of variance explained by each principal component (PC) is indicated on its corresponding axis.

The identified proteins were defined as variables in PCA analyses, in which FhNEJ stimulated samples formed compact clusters, whereas the rest of the samples displayed a less optimal clustering (Fig 2). However, separation among stimulated and control samples was clear in all cases, especially along the horizontal axis represented by the first principal component which constitutes the main source of variability, depicting that the stimulation of only 3 h is enough to trigger changes at the proteomic level in all studied compartments. This analysis suggests that FhNEJ undergo an orchestrated pattern of protein expression with a low degree of variability after establishing contact with MPSIEC. Such response would be important in the tegument of *F*. *hepatica* provided its dynamic composition and continuous adaptation to the changing demands of the juvenile parasite during its migration and developmental process [28]. On the other hand, changes in MPSIEC showed a higher intragroup variation compared with the compartments analyzed in FhNEJ, similar to what has been described in comparable studies [30].

The presence of transmembrane (TM) domains in proteins identified in the two fractions (membrane and cytosol) of MPSIEC was assessed in order to determine the effectiveness of the enrichment procedure of the two obtained fractions. Seventy-four cytosolic proteins were predicted to contain at least one TM domain in their structure, which represents a 5.43% of the proteins identified in this fraction, while 170 proteins within the membrane fraction were predicted to contain TM domains, representing a 12.77% of the identified proteins, a proportion similar to other studies in which membrane-enriched fractions of mammalian cells extracts were obtained (e.g., [31]). Additionally, the protein COX-IV (Uniprot ID P19783), used to prove the enrichment of membrane proteins (e.g., [32]) was detected only in the membrane fraction and not in the cytosol extract. This indicates the success of the enrichment procedure.

**Fig 3:**
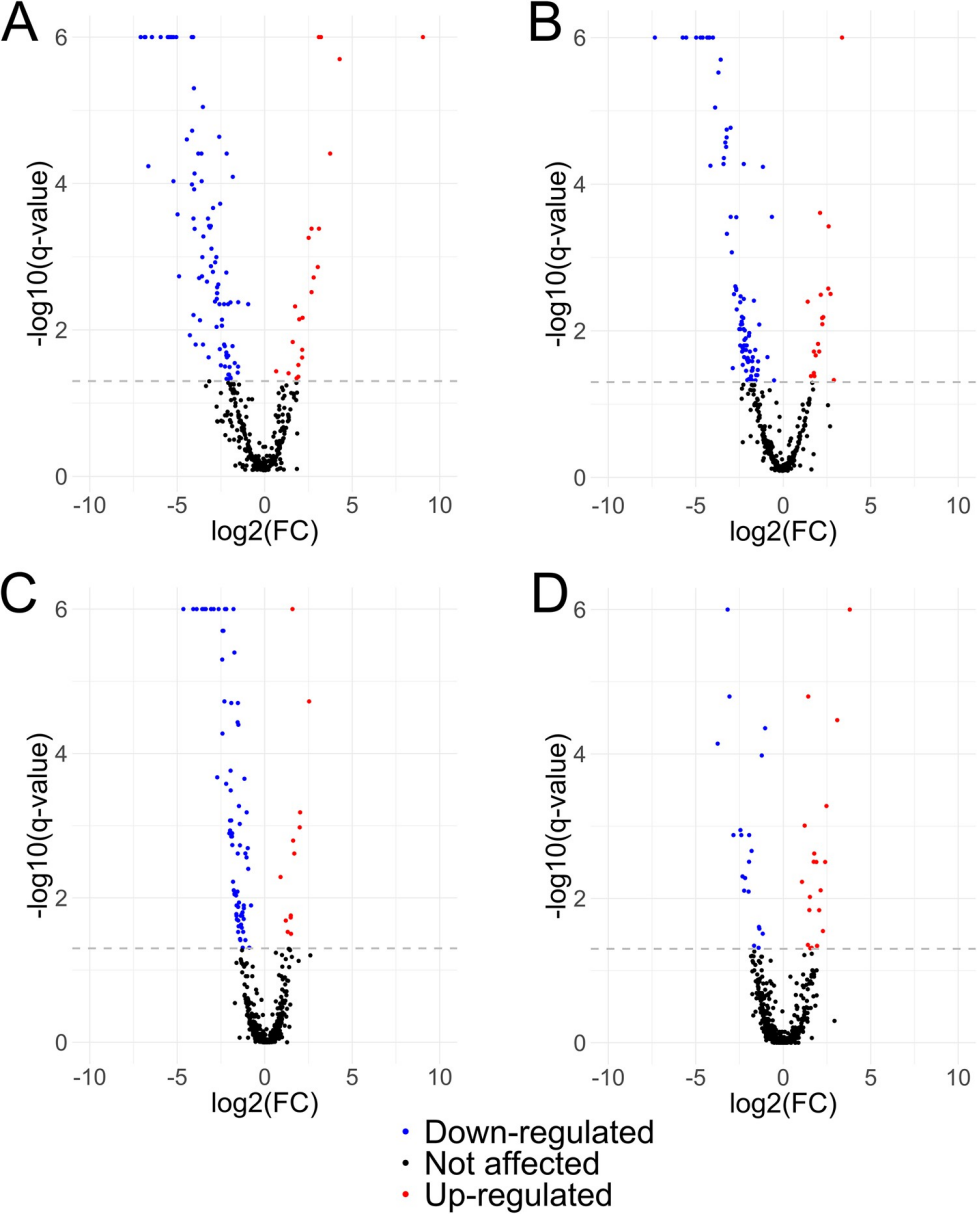
Volcano plots representing the changes in the proteomic profile of each group of samples analysed in the study. Volcano plots corresponding to FhNEJ detergent-soluble extract enriched with tegument (FhNEJ tegument; A), FhNEJ somatic (B), MPSIEC cytosol (C) and MPSIEC membrane (D) protein extracts. Red dots represent up-regulated proteins after stimulation challenge, and blue dots represent down-regulated proteins. Only DEPs with q-value <0.05 were considered as differentially expressed, and no fold change threshold was considered.

Differentially expressed proteins (DEPs) were assessed through Student’s t-test, and only DEPs with a q-value <0.05 were used for further analysis. Since fold change cutoffs are arbitrary and depend largely on the dynamic range of each technique, significancy of differentially abundant proteins was selected based only on q-value. A total of 109 DEPs meeting the above-mentioned criteria were found in the detergent-soluble extract enriched with tegument of FhNEJ (21 up- and 88 down-regulated) (Fig 3A), while 101 DEPs (17 up- and 84 down-regulated) were identified in the somatic extract (Fig 3B). Concerning MPSIEC extracts, a total of 93 DEPs (16 up- and 77 down-regulated) were identified among cytosolic proteins (Fig 3C), and 40 DEPs (20 up- and 20 down-regulated) were found in the membrane (Fig 3D). In sum, the number of DEPs from MPSIEC is 1.5 times lower than from FhNEJ, providing a similar proportion to that obtained in our previous *in vitro* interaction model after 24 h co-culture of FhNEJ with MPSIEC [14]. This ratio is also in line with a similar study in which dual-species RNA-Seq analysis of porcine intestinal epithelial cells upon co-culture with L3 larvae of *Ascaris suum* showed a low magnitude response by host cells after parasitic stimulation [3].

Our data also showed an imbalance towards down-regulated proteins, especially concerning FhNEJ extracts upon MPSIEC co-culture, which has been observed in similar studies (e.g., [33, 34]). This fact suggests that silencing of protein expression could be faster than its induction in FhNEJ after a short time of stimulation with host tissues, which could also be related to the turnover and/or shedding mechanisms that take place on the FhNEJ surface shortly after excystment as part of their immune evasion strategies [35, 36].

**Fig 4:**
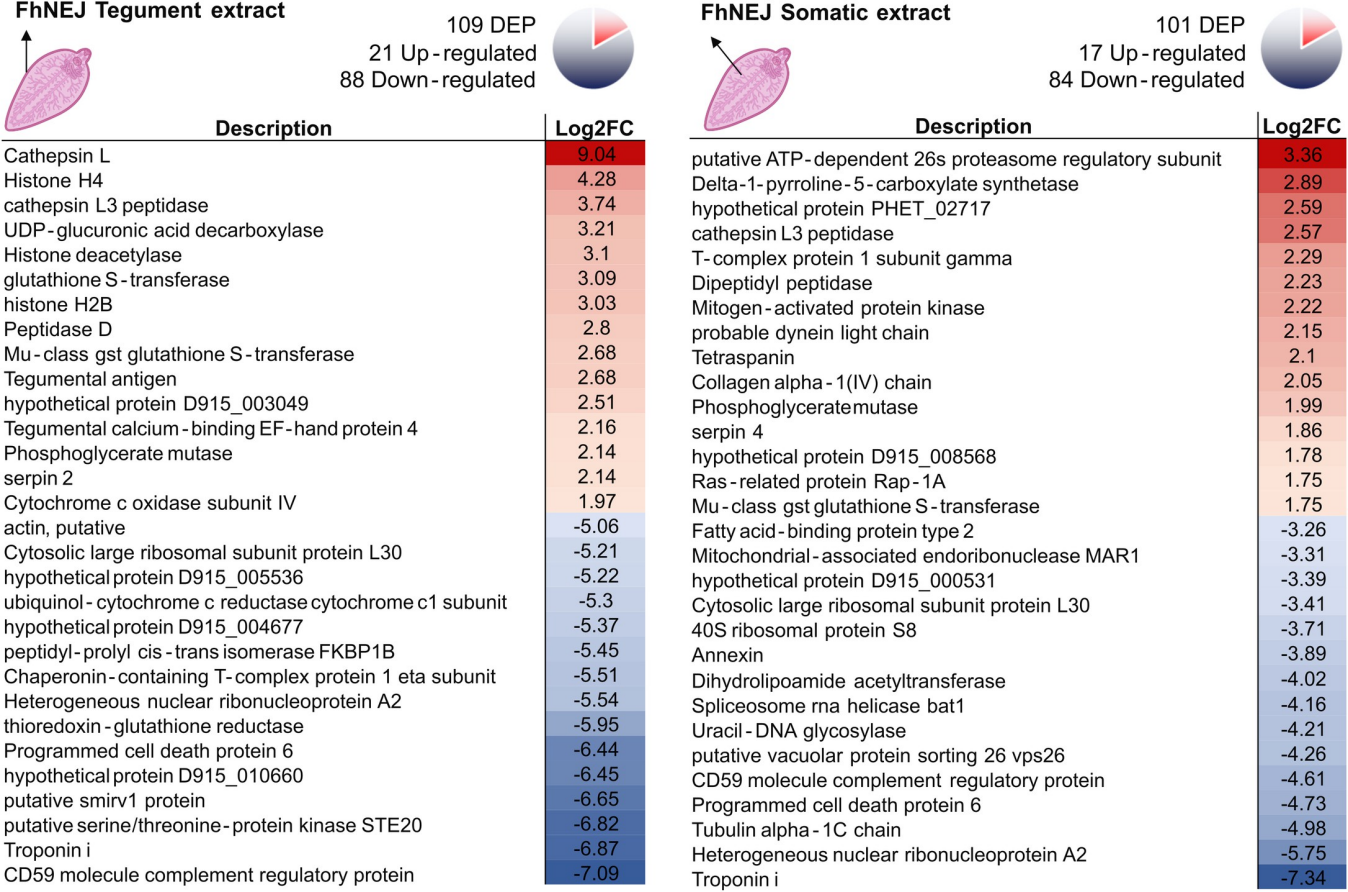
Differentially expressed proteins in FhNEJ after incubation with MPSIEC. The first 15 proteins with the highest (represented in red) or lowest (represented in blue) fold change within the FhNEJ detergent-soluble extract enriched with tegument (FhNEJ Tegument extract) and somatic extract (FhNEJ Somatic extract) are shown. Icons created with BioRender.com.

Up and down-regulated proteins in FhNEJ and MPSIEC were identified by database search and bioinformatic analysis (S1 Table for FhNEJ and S2 Table for MPSIEC). The top 15 regulated proteins with highest fold changes are shown in Figs 4 (FhNEJ) and 5 (MPSIEC).

**Fig 5:**
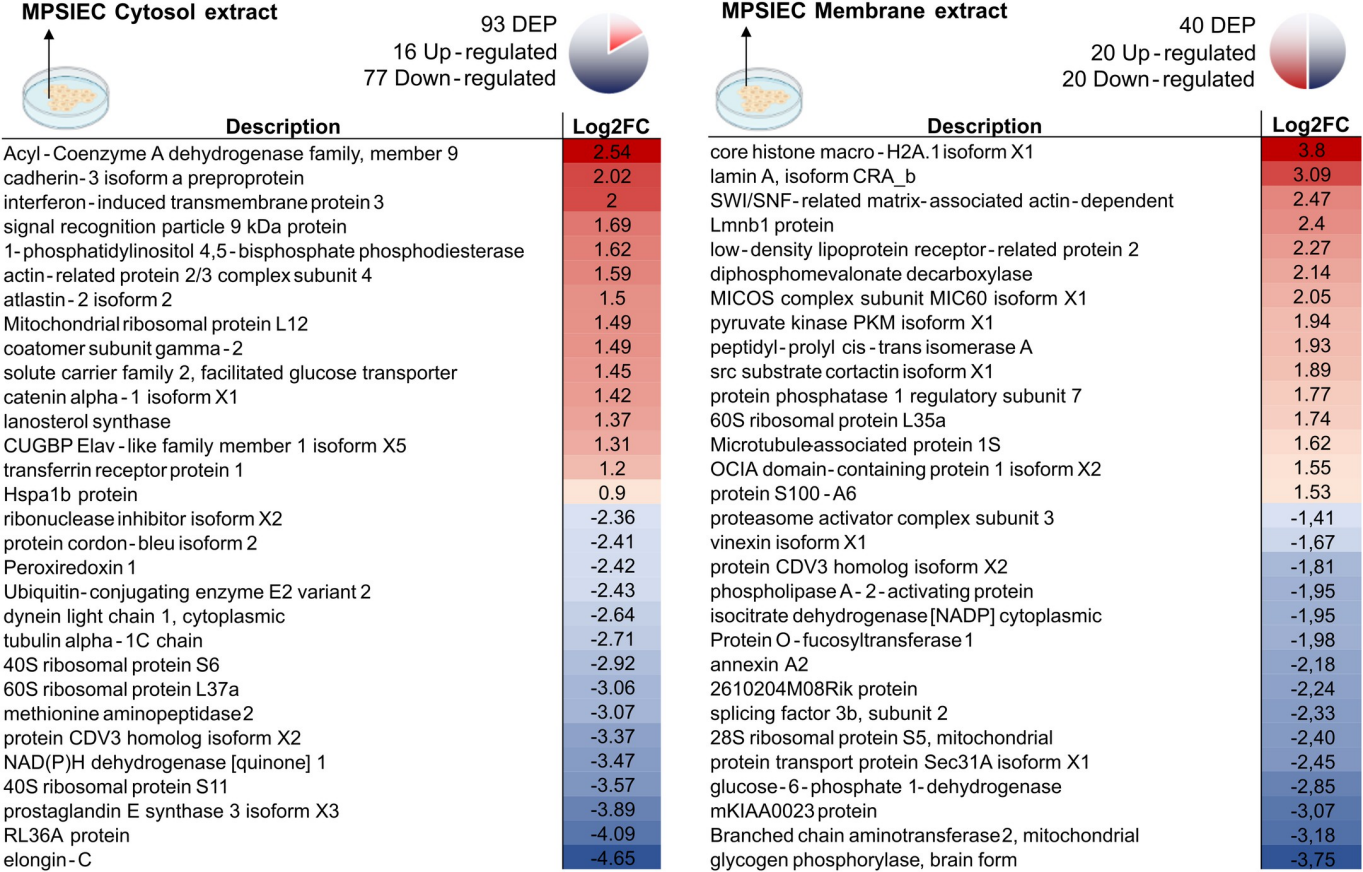
Differentially expressed proteins in MPSIEC after incubation with FhNEJ. The top 15 proteins with highest (represented in red) or lowest (represented in blue) fold change within the MPSIEC membrane and cytosol extracts are shown. Icons created with BioRender.com.

### Functional annotation

#### FhNEJ protein expression changes rapidly in response to interaction with the host intestinal epithelial barrier

**Fig 6:**
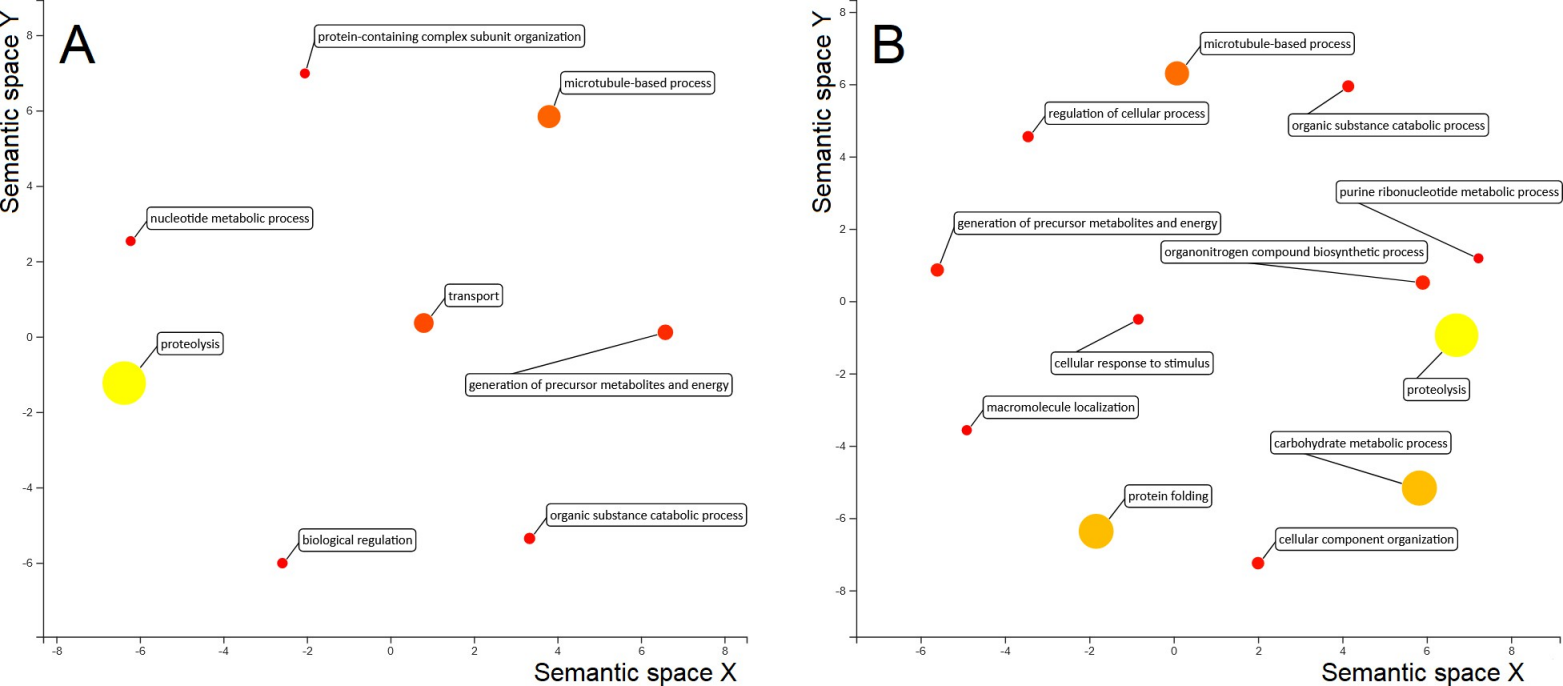
ReViGO plot showing the main GO terms within the Biological Process (BP) category in FhNEJ. The BP GO terms corresponding to up- and down-regulated proteins in the detergent-soluble extract enriched with tegument (A) and soma (B) extracts of FhNEJ after incubation with MPSIEC are shown. The size and colour of each circle represents the Nodescore of each GO term, and the spacing between circles refers to the similarity between the represented terms.

In the detergent-soluble extract enriched with tegument of FhNEJ, the top over-represented BPs were proteolysis, microtubule-based process and transport (Fig 6A), while the main MFs identified in this antigenic compartment were transferase activity, metal ion binding and oxidoreductase activity (S1 Fig).

Regarding proteolysis, a considerable number of proteomic studies have demonstrated the high level of protein complexity of the juvenile stages of *F*. *hepatica* in terms of proteolysis-related molecules [16,37,38]. This situation is not unexpected considering that this function is pivotal for the parasite invasion process. More specifically, trematode peptidases have been pointed out as key molecules in facilitating parasite migration, nutrition, immune evasion and other important host-parasite interactions [39]. Cathepsin peptidases are among the major proteolytic enzymes involved in FhNEJ virulence and tissue migration and they represent the most abundant proteases in the secretions of *F*. *hepatica* and a paradigmatic example of evolutionary divergence in response to selection pressure [40]. In fact, 23 and 11 different sequences of cathepsins belonging to the L and B classes have been identified in the *F*. *hepatica* genome [41], and their expression shows a stage-specific temporal shift that correlates with juvenile development and the organ invasion process [42,43].

The high abundance of proteases among the up-regulated proteins in the detergent-soluble extract enriched with tegument of FhNEJ upon incubation with MPSIEC is remarkable (S1 Table). These included cathepsin L (identified as cathepsin L4 (CL4) after comparison of its prosegment with those corresponding to *F*. *hepatica* consensus sequences of CL1 and CL5 prosegments [44]), cathepsin L3 peptidase (CL3) and peptidase D. The first and third upregulated proteins in FhNEJ tegument upon contact with host cells (CL4 and CL3, respectively) have been found in the transcriptome of FhNEJ axenically maintained *in vitro* for 1, 3 and 24 h [37], but only CL3, and not CL4, has been found in protein extracts of FhNEJ tegument [17]. Our results suggest that CL4 would be overexpressed in the detergent-soluble extract enriched with tegument of FhNEJ only when subjected to interact with host cells. On the contrary, CL3 would be expressed in FhNEJ after *in vitro* excystment without requiring host stimulus. Thus, CL4 may play a role at the host-parasite interface upon contact of FhNEJ with the host. Importantly, CL3 is known for its collagenolytic activity, which is especially efficient in digesting type I and II collagens [45]. Provided that FhNEJ must overcome four layers in the small intestine wall (mucosa, submucosa, muscularis externa and serosa) that are highly rich in collagen [46] in order to reach the peritoneum, the over-expression of proteases with collagenolytic activity in the host-parasite interface could be crucial for parasite invasion. Moreover, this important function might be followed by the activity of other up-regulated proteases such as peptidase D (also known as prolidase), whose most studied substrates are dipeptides that are generated during collagen breakdown [47]. Together with these proteases, the protease inhibitor serpin 2 is also upregulated in this compartment of FhNEJ after incubation with MPSIEC. Serpin 2, characterized as a surface molecule in this parasite, can both protect the FhNEJ from host proteolysis and play a role in immune evasion [48].

Interestingly, some proteins traditionally associated with the nucleus such as histones H4 and H2B and histone deacetylase were also up-regulated in the FhNEJ detergent-soluble extract enriched with tegument after co-incubation with host intestinal epithelial cells. These proteins have important functions in chromosomic DNA organization [49] and they can also be released to the cytosol and the extracellular space to perform other functions, such as antimicrobial responses [50]. In fact, it has been demonstrated that histones (e.g. H4 or H2B) and their derivatives, which are cleaved from histones by cathepsins [51], directly eliminate a wide range of pathogens in a similar way to other antimicrobial peptides [50]. This mechanism, by which proteins exhibit ‘unexpected’ functions separate from their canonical activities, is referred to as ‘moonlighting’. Moonlighting is directly linked to unconventional protein secretion routes that allow for the translocation of cytosolic antigens to the host-parasite interface [52]. This is also the case of surface-located glycolytic enzymes, such as phosphoglycerate mutase, which is up-regulated in the tegument and the soma of FhNEJ in this study. This enzyme normally participates in energy metabolism, but when located extracellularly it also plays a role in parasite invasion and establishment within the host [53].

Potentially linked to immune regulation, we found glutathione-S-transferase (GST) and the calcium-binding EF-hand protein 4 (CABP4) to be overexpressed in this protein fraction of FhNEJ upon incubation with MPSIEC. *F*. *hepatica* GST (also found here overexpressed in the somatic extract of FhNEJ) has shown anti-inflammatory properties [54], and *F*. *gigantica* CABP4 stimulated IL-2 and IFN-γ Th1-type cytokines and reduced IL-10 (Th2) expression levels by activated goat monocytes [55]. As observed in other trematode species (e.g., schistosomes [56]), these proteins could play a role in the first steps of infection by protecting FhNEJ from inflammatory responses as well as eliciting a transient Th1-type immune response during early infection.

Down-regulated proteins in the detergent-soluble extract enriched with tegument of the parasite include components of the muscle contraction machinery (troponin, actin, dynein, titin, etc.) and proteins involved in metabolite transport (fatty acid binding protein, vesicle-associated membrane protein-associated protein A) along with other multi-purpose proteins (annexin, peptidyl-prolyl cis-trans isomerase). Noteworthy, some biological processes with up-regulated proteins (i.e. proteolysis or antioxidant response) also exhibit down-regulated representatives, and might therefore be finely regulated during the early phases of *F*. *hepatica* infection. This could be the case of the above-mentioned cathepsins. In fact, the results obtained in this study showed a down-regulation in the expression of two isoforms of cathepsin B after contact of FhNEJ with MPSIEC. FhCB peptidases appear to be highly expressed by FhNEJ and down-regulated in adult flukes [40, 57], although defined isoforms of CBs could have important roles in the excystment process but not in the subsequent migration of FhNEJ. Overall, these results suggest an important role of the interaction between FhNEJ and the host intestinal wall in the cathepsin expression shift observed in *F*. *hepatica* in correlation to parasite development and migration through host tissues.

Interestingly, the downregulated protein with the highest Log2FC in this FhNEJ extract, also corresponding to the fifth most downregulated protein in somatic extracts of FhNEJ upon MPSIEC incubation, is CD59, a complement regulatory protein that protects cells from the destructive action of the complement system. Homologue CD59 proteins have been previously described to be expressed and secreted by FhNEJ *in vitro* [37, 58], together with other parasite proteins that could impair the host’s complement response, such as glyceraldehyde 3-phosphate dehydrogenase (GAPDH) [37, 38, 58], which is similarly downregulated in the tegument extract of FhNEJ upon incubation with MPSIEC. Thus, it is possible that the down-regulation of CD59-like molecule and GAPDH herein identified could be an indication of depletion of these molecules in the tegument compartment as a result of increased secretion upon interaction with the host to counteract complement attack. However, it is also known that FhNEJ employ multiple mechanisms to inhibit complement attack during invasion [59], so alternative strategies to avoid this host defence mechanism could also be taking place.

In the somatic FhNEJ extract, the main GO terms in the BP category were proteolysis, carbohydrate metabolic process, protein folding and microtubule-based process (Fig 6B), and transferase activity, peptidase activity and metal ion binding in the MF category (S1 Fig). Up-regulated proteins in this compartment exhibited a profile similar to that described in the tegument, including proteins involved in peptide degradation (CL3, dipeptidyl peptidase), antioxidant response (GST) or metabolic enzymes (phosphoglycerate mutase), as well as proteins involved in signalling cascades (Mitogen-activated protein kinase) or cell-cell adhesion (tetraspanin). Tetraspanins are transmembrane proteins involved in important functions linked with the maintenance of the integrity of the adult fluke tegument [60]. Noteworthy, tetraspanins from other helminth trematodes such as *Schistosoma mansoni* or *Opistorchis viverrini* have elicited promising results as vaccine candidates against schistosomiasis and opisthorchiasis, respectively [61, 62]. However, the biological role of the identified up-regulation of this protein in the internal tissues of the FhNEJ should be further investigated.

Down-regulated proteins in the soma of FhNEJ included proteins also down-regulated in their tegument (fatty acid binding protein, GAPDH, peptidyl-prolyl cis-trans isomerase). Additionally, legumain like and leucyl aminopeptidase, superoxide dismutase, transketolase and pyruvate dehydrogenase, representing functions similar to those down-regulated in the tegument of FhNEJ, were also found as down-regulated in the somatic extracts of FhNEJ.

Regarding antioxidant enzymes, our data revealed that the expression of four isoforms of one of their main representatives, GST, is up-regulated within the FhNEJ somatic and tegument extracts after co-culture with MPSIEC. It has been described that GSTs account for 4% of *F*. *hepatica* excretory/secretory products and that these enzymes have a key role in detoxifying both endogenous and exogenous toxins arising from the oxidative host defence response [63]. For that reason, this parasitic antigen has been proposed as a vaccine candidate against fasciolosis [64]. However, our data also revealed the presence of a GST isoform that is down-regulated within the FhNEJ somatic extract. This particular shift in the expression pattern of parasitic isoforms of the same protein upon interaction with host cells could be explained by finely regulated biochemical redundancy mechanisms, which would hamper the deleterious effects of the immune response against these specific antigens. This bidirectional regulation of molecules that are important for parasite survival and development could also explain the reported high variability in protection rates and the partial success of vaccination studies against fasciolosis that included these diversely-regulated antigens in their formulations [10,65].

The comparison of the results of the present analysis with that performed before by our group by using the iTRAQ methodology after 24 h co-incubation of FhNEJ and MPSIEC [14], shows that proteolysis seems to play a key role in FhNEJ after co-culture with MPSIEC, at both 3h and 24h post-incubation (pi), as this functional category is up-regulated at both times. However, the protease profile shows some differences between the two incubation times, as cathepsin L3 is the major isoform together with peptidase D after 3h pi, whereas at 24h pi cathepsin L1 and peptidase C1 are up-regulated and cathepsins L3, L4 and B, along with papain and legumain, are down-regulated. This suggests that at 24h pi the parasite is already expressing adult stage specific isoforms, while juvenile isoforms are already decaying. Regarding regulation of proteolysis, this seems to play a more important role in the earliest stages (3h pi), as serpin 2 is up-regulated at this time, while cystatin is found as down-regulated at 24h pi. Similarly, response to oxidative stress is represented by several GST isoforms that are up-regulated at 3h pi but not at 24h pi.

On the other hand, muscle contraction and cytoskeleton reorganization processes seem to be more represented at 24h pi, as at this time several related proteins are found to be up-regulated while they are not found at 3h pi, including dynein, cadherin, myophilin and catenin alpha. Several metabolic enzymes could be also differentially expressed at both times pi. The landscape at 3h pi is mainly aerobic (including several up-regulated respiratory enzymes such as cytochrome c oxidase and quinone oxidoreductase), while at 24h pi it seems to evolve to an anaerobic profile with a greater abundance of glycolytic enzymes (e.g., aldolase, GAPDH). Interestingly, histone isoforms are found as up-regulated proteins at both times pi, with histone H4 present at both times, while histone H2B is found at 3h and histone H2A at 24h. In summary, comparison of the results obtained here at 3h pi obtained by SWATH and the previous results at 24 h pi obtained by iTRAQ, show that proteolysis and its regulation, muscle contraction and metabolism could be differentially regulated in FhNEJ upon MPSIEC co-incubation at different times pi. Nevertheless, this should be further explored in more comparable studies, since the methods used to check up- and down-regulated proteins in FhNEJ after co-culture with host MPSIEC between the two above-mentioned studies are different regarding the proteomic and statistical approaches.

#### Stimulation of MPSIEC with FhNEJ results in the modulation of protein expression in host cells

In vertebrate animals, the small intestine is the organ with the largest surface area in contact with the outside environment. Although its main function is the absorption of water and nutrients from the diet, it is also the entry point for a multitude of pathogens, including foodborne parasites [66]. As a matter of fact, intestinal epithelial cells are considered the first line of defence against pathogens, since they can detect them and trigger appropriate responses by underlying immune cells in order to maintain intestinal homeostasis [67,68]. However, the mechanisms by which the host intestinal epithelium detects parasitic invasion and stimulates appropriate immune responses is still a matter of debate [69].

**Fig 7:**
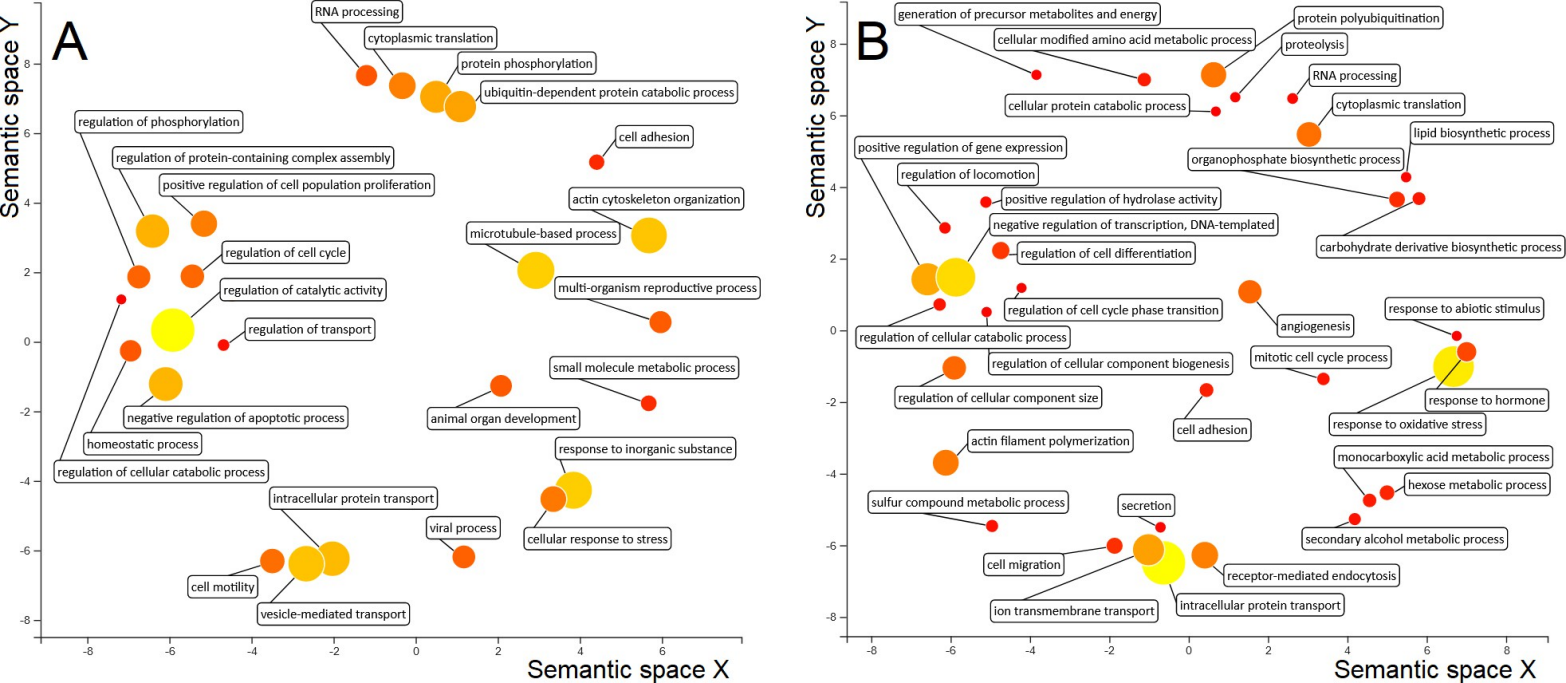
ReViGO plot showing the main GO terms within the Biological Process (BP) category in MPSIEC. The BP GO terms corresponding to up- and down-regulated proteins in the cytosol (A) and membrane (B) extracts of MPSIEC after incubation with FhNEJ are shown. The size and colour of each circle represents the Nodescore of each GO term, and the spacing between circles refers to the similarity between the represented terms.

Our proteomics approach showed a considerable number of altered processes that were identified in the extracts of MPSIEC upon interaction with FhNEJ. In particular, some of the main modified BPs in MPSIEC cytosolic extracts were regulation of catalytic activity, actin cytoskeleton organisation, response to inorganic substance and vesicle-mediated transport (Fig 7A), whereas the membrane fractions showed differential expression of proteins involved in intracellular protein transport, response to oxidative stress and negative regulation of transcription (Fig 7B). Within the MF category, the most represented GO terms were RNA binding, structural constituent of ribosome and protein-containing complex binding among cytosolic proteins, and protein-containing complex binding, RNA binding and actin binding in the membrane extracts (S2 Fig).

Over-expressed proteins in the cytosol of mouse cells were involved in functions such as vesicle transport (coatomer subunit gamma-2, atlastin-2) and lipid biosynthesis (acyl-Coenzyme A dehydrogenase family member, lanosterol synthase) along with proteins involved in the response to stress (Hspa1b protein) or viruses (interferon-induced transmembrane protein 3 (IFITM3)). Among down-regulated proteins, there was an abundance of ribosomal constituents together with cytoskeleton-related proteins. Moreover, our results revealed that the expression of pro-inflammatory mediators (prostaglandin E synthase 3 (PTGES3)) and peptides with antimicrobial activity (Lysophospholipase 1) was also decreased.

Up-regulated proteins within the MPSIEC membrane extracts included nuclear lamin components (lamin A, CRA_b isoform, Lmnb1 protein), chromatin-interacting proteins (SWI/SNF-related matrix-associated actin-dependent regulator of chromatin, chromobox protein homolog 1) and enzymes involved in energy metabolism (diphosphomevalonate decarboxylase, pyruvate kinase PKM). Down-regulation of proteins related to carbohydrate catabolism (glycogen phosphorylase, glucose-6-phosphate 1-dehydrogenase, cytoplasmic isocitrate dehydrogenase (NADP)) or response to pathogens (phospholipase A-2-activating protein (PLAA), annexin A2) was observed in this extract.

Taken together, these results suggest a potential modulation of MPSIEC immune responses by FhNEJ via the regulation of important host defence mediators. These mechanisms could have a crucial role not only in relation to the overall success of parasite invasion but also in the outcome of concomitant infections. Particularly, phospholipase A-2 plays a key role in the initiation, propagation and resolution of inflammation at the intestinal level. This enzyme is considered one of the main components of the innate immune system of the intestinal epithelial barrier against invading microbes due to its bacteriolytic properties [70]. Likewise, prostaglandin E2 is a principal mediator of inflammation and its role in mucosal defence is of paramount importance to maintain the integrity of the gastrointestinal tract [71]. Accordingly, down-regulation of host PTGES3 and PLAA in response to FhNEJ stimulation could potentially facilitate FhNEJ invasion as well as bacterial colonization of the intestinal epithelium. This interspecies interaction would be in line with an increased risk of concomitant bacterial infections that has been reported in fasciolosis [72].

The stimulation of MPISEC with FhNEJ also resulted in the upregulation of IFITM3, an interferon-induced protein that is also constitutively expressed by virtually all human and mouse cell types. IFITM3 restricts cellular entry of enveloped viruses, including SARS coronaviruses [73], by either accelerating endosomal acidification towards a degradative state or by altering the mechanical properties and composition of lipid bilayers, which disfavours the fusion between virus and target cell membranes [74]. This finding opens up a new perspective related to the potential of defined FhNEJ-derived proteins to modulate the susceptibility to viral infections [75].

Notwithstanding, and although a number of proteins with potential immune-related functions have been identified, further experiments aimed at testing the ability of MPSIEC exposed to FhNEJ to respond to specific Th1-inducing ligands should be performed in the future to clarify the immunomodulatory capacity of the FhNEJ in this model.

#### Protein association analysis reveals an orchestrated reduction in host ribosome function after co-culture with FhNEJ

**Fig 8:**
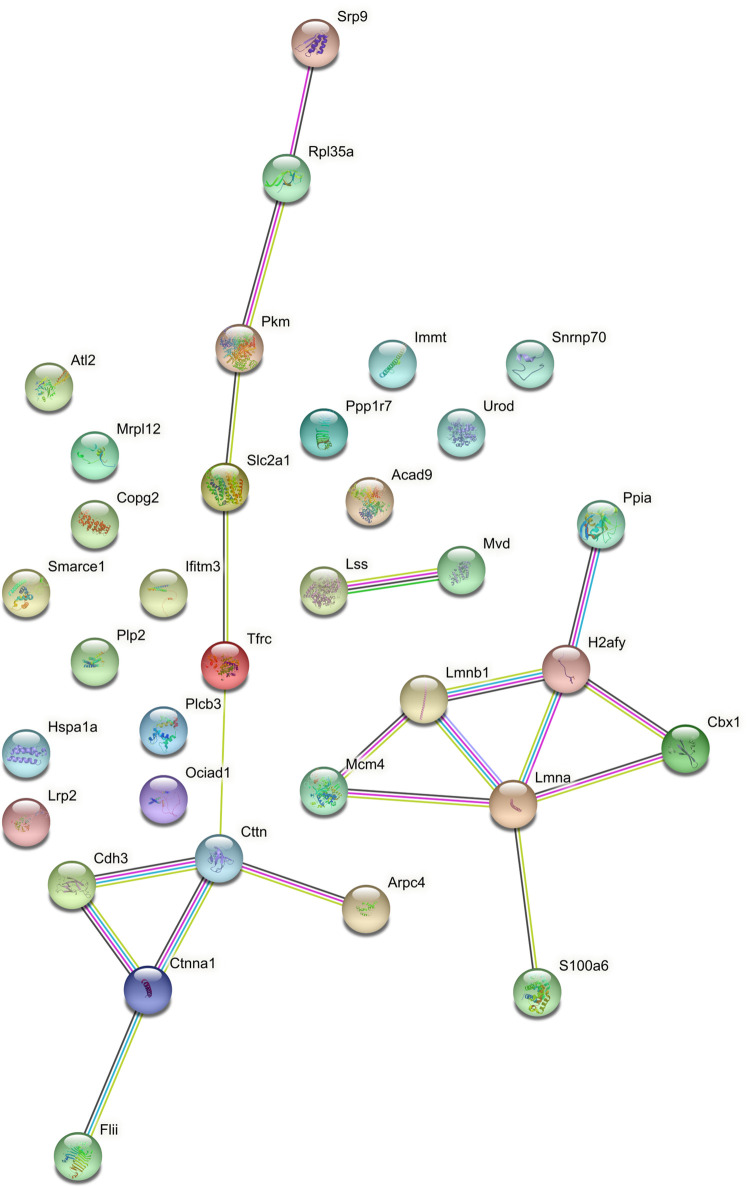
Interaction plot (STRING) showing protein-protein relations within the set of up-regulated MPSIEC proteins. Edges between network nodes represent protein-protein associations, which are classified as “known interaction” (light blue: from curated databases; purple: experimentally determined), “predicted interactions” (green: gene neighbourhood; red: gene fusions; blue: gene co-occurrence) or “others” (yellow: textmining; black: co-expression; pale blue: protein homology) (https://version-11-5.string-db.org/cgi/network?networkId=blUqyaKyKA9R).

The sum of up-regulated proteins in both compartments of MPSIEC was further analysed for interactions using STRING in order to establish possible protein-protein relationships that could be relevant to *F*. *hepatica* infection (Fig 8). This set presented a more diffuse interaction network with three clusters of association. One of them is characterized by the up-regulation of membrane proteins, specifically of components of the nuclear lamina (lamin A isoform CRA_b and Lmnb1 protein). Lamin A, B and C, also known as the nuclear intermediate-filament proteins, provide a scaffold for the nuclear envelope and protect the chromatin from physical damage [76]. The second association cluster of MPSIEC up-regulated proteins upon co-culture with FhNEJ is formed by an isoform of cadherin-3, catenin alpha-1 isoform X1, src substrate cortactin isoform X1, protein flightless-1 homolog isoform X1 and an actin-related protein. The association between cadherins and catenins creates a complex that is linked to the underlying actin cytoskeleton and involved in the expansion and completion of cell-cell adhesion processes, among others [77]. The up-regulation of these proteins could be part of a host defence mechanism aimed at increasing the adhesion properties of the epithelial cell barrier and play a role in cadherin-mediated host-parasite interactions, which have been described to be involved in tissue invasion by different pathogens [78]. The third cluster of host up-regulated proteins is represented by proteins involved in cholesterol biosynthesis (diphosphomevalonate decarboxylase and lanosterol synthase). Parasites are capable of exploiting cholesterol metabolism to impair the host immune response and also make use of this pathway for invasion purposes, since they employ cholesterol rich domains as an assembly platform for invasion [79].

**Fig 9:**
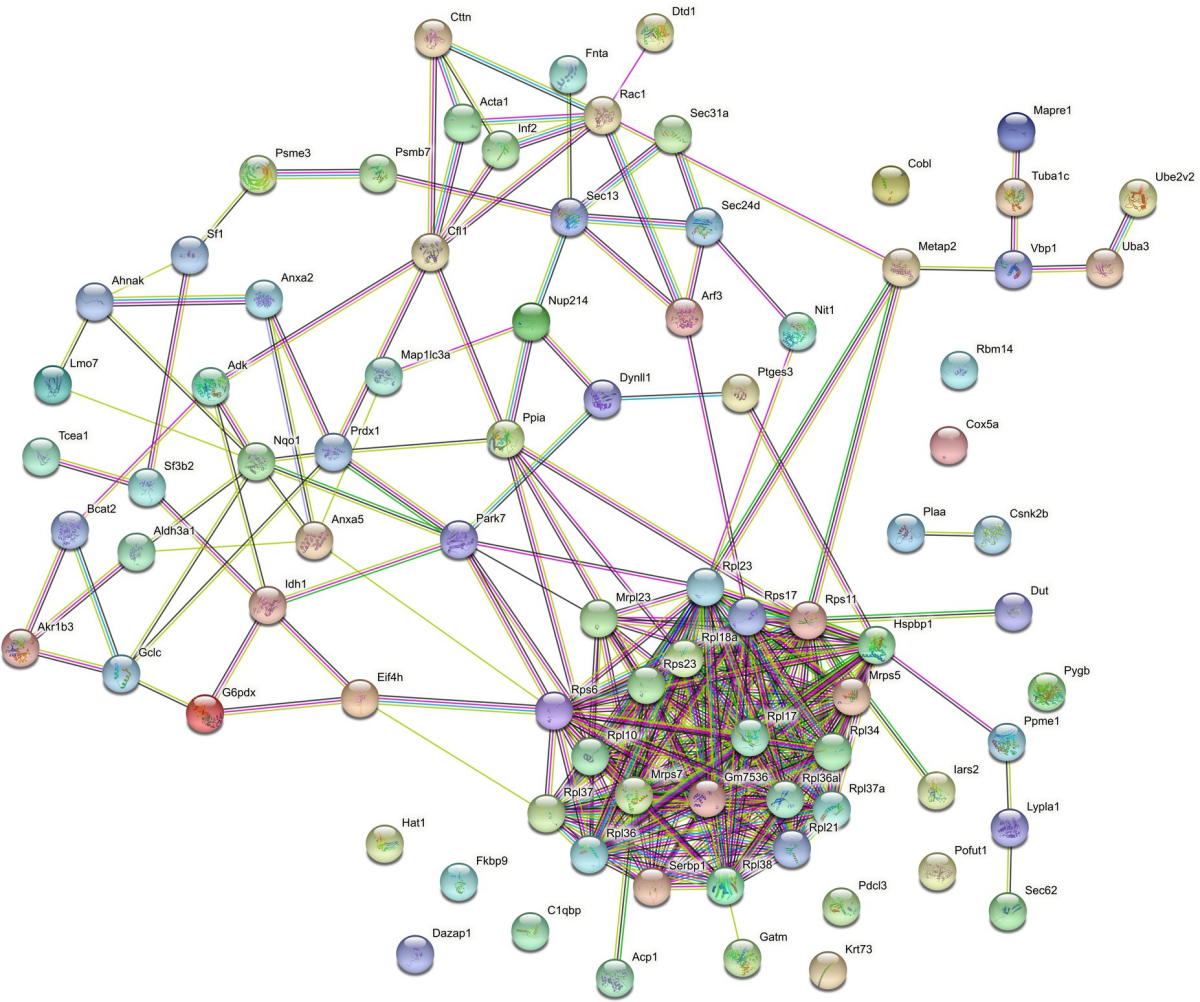
Interaction plot (STRING) showing protein-protein relationships within the set of down-regulated MPSIEC proteins. Edges between network nodes represent protein-protein associations, which are classified as “known interaction” (light blue: from curated databases; purple: experimentally determined), “predicted interactions” (green: gene neighbourhood; red: gene fusions; blue: gene co-occurrence) or “others” (yellow: textmining; black: co-expression; pale blue: protein homology) (https://version-11-5.string-db.org/cgi/network?networkId=bEnbwSoK41Pk).

The same analysis was performed for MPSIEC down-regulated proteins upon interaction with FhNEJ (Fig 9), and we detected a prominent cluster of ribosomal proteins as the most representative feature. Moreover, we found associations of these proteins with other important representatives, such as Hsp70-binding protein 1, plasminogen activator inhibitor 1 RNA-binding protein isoform X2 and protein/nucleic acid deglycase DJ-1. In addition to these interactions, additional proteins were detected as interactors in smaller networks, such as cofilin-1, peroxiredoxin-1, or protein SEC13 homolog. A downstream analysis of MPSIEC down-regulated proteins using the Cytoscape MCODE package also detected the abovementioned cluster of ribosomal proteins, and annotation with DAVID revealed that the functions involving this protein family include translation, ribosomal small subunit biogenesis and ribosomal assembly. Intriguingly, a similar reduced ribosome function in intestinal epithelial cells was observed in an analogous *in vitro* model carried out to study host-parasite interactions in porcine ascariosis [3]. Ribosomes are not only essential constituents of the protein synthesis machinery but can also be involved in the so-called extra-ribosomal functions, which include regulation of cell growth and proliferation, differentiation, apoptosis, and DNA repair [80]. Additionally, down-regulation of ribosome function has been reported in response to various types of stress, such as cell cycle arrest and apoptosis [81], so the down-regulation of ribosomal function herein described could be representative of a general loss of homeostasis in the host upon infection with *F*. *hepatica*.

## Conclusions

After ingestion of infective metacercariae, FhNEJ are released in the host duodenum and start invading host tissues by crossing the intestinal epithelial barrier in a finely regulated process that takes place in 2–3 h and represents the first direct contact between *F*. *hepatica* and its host. In this work, we have set up an *in vitro* model that replicates this important stage of the infection process by co-culturing FhNEJ and MPSIEC. The use of SWATH-MS to analyze and quantify the dynamic changes in the protein repertoire of both the parasite and the host after co-stimulation has allowed us to identify a set of proteins and processes that shed light into the intricate molecular crosstalk that occurs between both organisms. The obtained results show rapid changes in the protein expression pattern of FhNEJ in response to interaction with the host intestinal epithelium, including proteolytic, antioxidant and immunoregulatory differentially expressed proteins. MPSIEC also responded to parasite stimulus by showing alterations in the expression of proteins related to immunomodulation and cell-cell interactions, as well as a remarkable decrease in ribosome function. Future studies aimed at confirming the functional consequences of host-parasite interactions at this stage of infection could help us define new targetable candidates for an efficient elimination of *F*. *hepatica* during early fasciolosis.

## Supporting information

S1 Fig ReViGO plot showing the main GO terms within the Molecular Function (MF) category in FhNEJ. The MF GO terms corresponding to up- and down-regulated proteins in the detergent-soluble extract enriched with tegument (A) and soma (B) extracts of FhNEJ are shown. The size and colour of each circle represents the Nodescore of each GO term, and the spacing between circles refers to the similarity between the terms represented.
(JPG)

S2 Fig ReViGO plot showing the main GO terms within the Molecular Function (MF) category in MPSIEC. The MF GO terms corresponding to up- and down-regulated proteins in the cytosol (A) and membrane (B) extracts of MPSIEC are shown. The size and colour of each circle represents the Nodescore of each GO term, and the spacing between circles refers to the similarity between the terms represented.
(JPG)

S1 Table Quantitative and annotation data of the differentially expressed proteins within the FhNEJ fractions. Uniprot accession codes were assigned as the match with the highest score in a BLAST search against the *F*. *hepatica* sequences available in this database (downloaded on 25 February 2020), whereas the remaining data were obtained during the Blast2GO annotation pipeline described previously.
(XLSX)

S2 Table Quantitative and annotation data of the differentially expressed proteins within the MPSIEC fractions. Data were obtained during the Blast2GO annotation pipeline described previously.
(XLSX)

S1 Video Footage showing the *in vitro* host-parasite interaction model developed in this study. In the video, a single FhNEJ can be observed crawling over the MPSIEC monolayer with a characteristic piston movement.
(MOV)
