# Supplementary figures and images for: Proteomics coupled with in vitro model to study the early crosstalk occurring between newly excysted juveniles of Fasciola hepatica and host intestinal cells
Source: PLoS Negl Trop Dis. 2022 Oct 12;16(10):e0010811. doi: 10.1371/journal.pntd.0010811 (PMC9555655; doi:10.1371/journal.pntd.0010811)

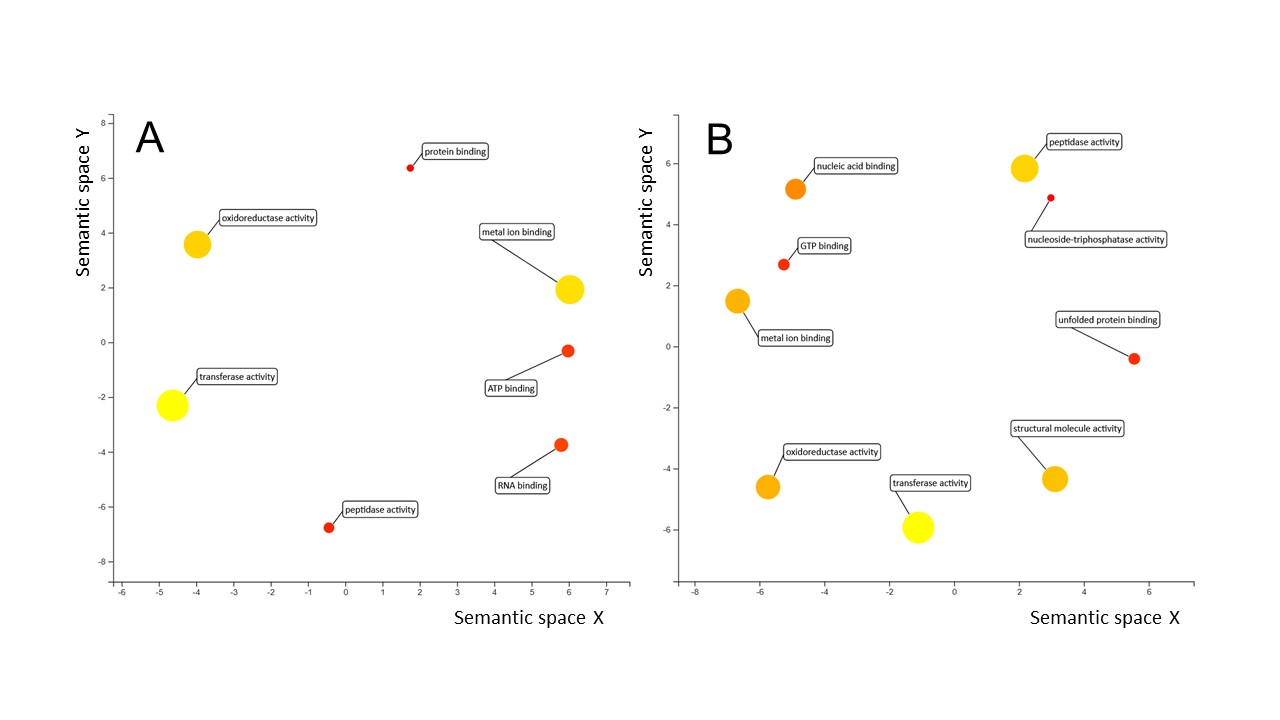

Supplement: S1 Fig — The MF GO terms corresponding to up- and down-regulated proteins in the detergent-soluble extract enriched with tegument (A) and soma (B) extracts of FhNEJ are shown. The size and colour of each circle represents the Nodescore of each GO term, and the spacing between circles refers to the similarity between the terms represented. (JPG) [file pntd.0010811.s001.JPG]

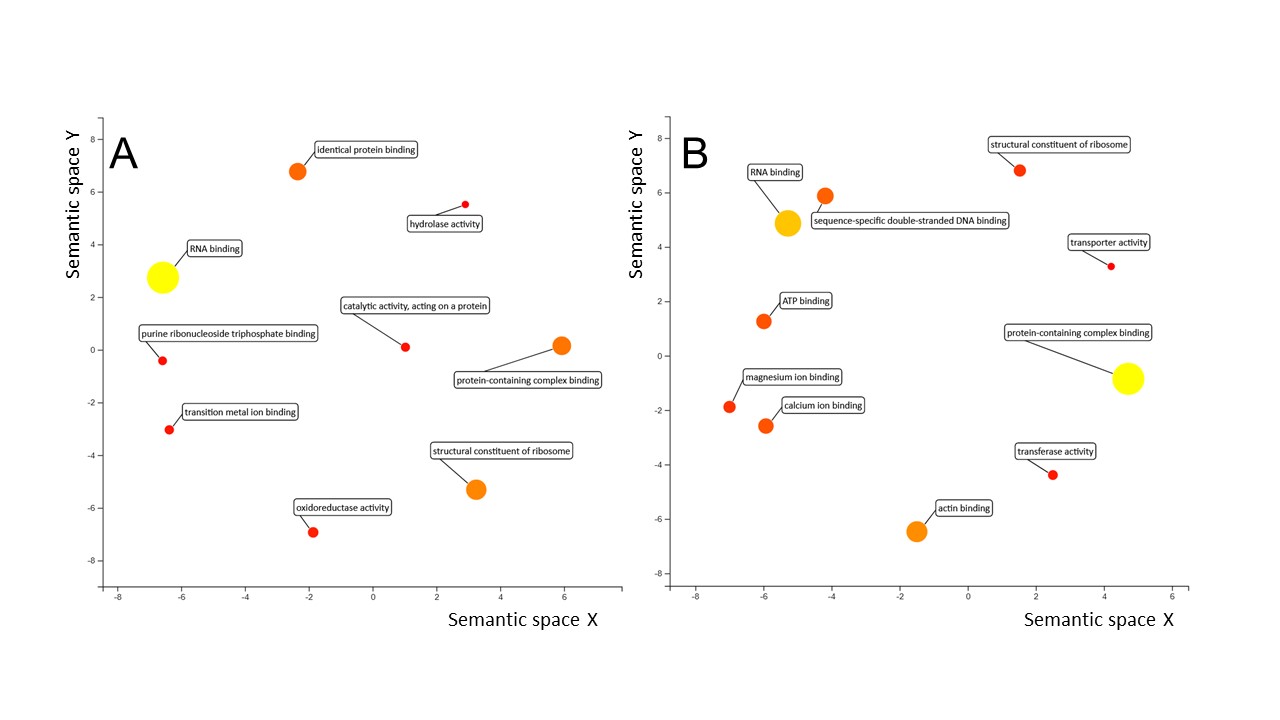

Supplement: S2 Fig — The MF GO terms corresponding to up- and down-regulated proteins in the cytosol (A) and membrane (B) extracts of MPSIEC are shown. The size and colour of each circle represents the Nodescore of each GO term, and the spacing between circles refers to the similarity between the terms represented. (JPG) [file pntd.0010811.s002.JPG]
